# Diagnosis, prevalence, and mortality of sarcopenia in dialysis patients: a systematic review and meta‐analysis

**DOI:** 10.1002/jcsm.12890

**Published:** 2022-01-05

**Authors:** Xiaoyu Shu, Taiping Lin, Hui Wang, Yanli Zhao, Tingting Jiang, Xuchao Peng, Jirong Yue

**Affiliations:** ^1^ Department of Geriatrics, West China Hospital Sichuan University Chengdu Sichuan China; ^2^ National Clinical Research Center for Geriatrics, West China Hospital Sichuan University Chengdu Sichuan China

**Keywords:** Sarcopenia, Dialysis, Diagnosis, Prevalence, Mortality

## Abstract

There is no consensus on the prevalence of sarcopenia or its impact on mortality in end‐stage renal disease patients undergoing dialysis. This review aimed to summarize the diagnostic criteria of sarcopenia and its prevalence and impact on the mortality of end‐stage renal disease patients undergoing dialysis. Embase, MEDLINE, PubMed, and Cochrane Library were searched from inception to 8 May 2021 to retrieve eligible studies that assessed muscle mass by commonly used instruments, such as dual‐energy X‐ray absorptiometry, bioelectrical impedance analysis, magnetic resonance imaging, and body composition monitor. Two assessment tools matched to study designs were employed to evaluate study quality. Pooled sarcopenia prevalence was calculated with 95% confidence interval (CI), and heterogeneity was estimated using the *I* ^2^ test. Associations of sarcopenia with mortality were expressed as hazard ratio (HR) and 95% CI. The search identified 3272 studies, and 30 studies (6162 participants, mean age from 47.5 to 77.5 years) were analysed in this review. The risk of bias in the included studies was low to moderate. Twenty‐two studies defined sarcopenia based on low muscle mass (LMM) plus low muscle strength and/or low physical performance, while eight studies used LMM alone. Muscle mass was assessed by different instruments, and a wide range of cut‐off points were used to define LMM. Overall, sarcopenia prevalence was 28.5% (95% CI 22.9–34.1%) and varied from 25.9% (*I* ^2^ = 94.9%, 95% CI 20.4–31.3%; combined criteria) to 34.6% (*I* ^2^ = 98.1%, 95% CI 20.9–48.2%; LMM alone) (*P* = 0.247 between subgroups). The statistically significant differences were not found in the subgroups of diagnostic criteria (*P* > 0.05) and dialysis modality (*P* > 0.05). Additionally, the sarcopenia prevalence could not be affected by average age [regression coefficient 0.004 (95% CI: −0.005 to 0.012), *P* = 0.406] and dialysis duration [regression coefficient 0.002 (95% CI −0.002 to 0.005), *P* = 0.327] in the meta‐regression. The pooled analyses showed that combined criteria of sarcopenia were related to a higher mortality risk [HR 1.82 (*I* ^2^ = 26.3%, 95% CI 1.38–2.39)], as was LMM [HR 1.61 (*I* ^2^ = 26.0%, 95% CI 1.31–1.99)] and low muscle strength [HR 2.04 (*I* ^2^ = 80.4%, 95% CI 1.19–3.5)]. Although there are substantial differences in diagnostic criteria, sarcopenia is highly prevalent in dialysis patients and is linked to increased mortality. The standardization of sarcopenia diagnostic criteria would be beneficial, and future longitudinal studies are needed to investigate the prevalence and prognostic value of sarcopenia in dialysis patients.

## Introduction

With the development of renal replacement therapies, including peritoneal dialysis (PD), haemodialysis (HD), kidney transplantation, and continuous renal replacement therapy (which is often used for acute renal failure), patients with end‐stage renal disease (ESRD) are routinely choosing treatment by PD, HD, and kidney transplantation to prolong their lifespan.^1^ Because of the shortage of donor kidneys, PD and HD are increasingly chosen by patients with ESRD.

Existing literature suggests that sarcopenia is common among ESRD patients undergoing dialysis. Firstly, metabolic disorders and inflammation induced by kidney failure result in the development of sarcopenia. The former include nutritional deficiency, insulin resistance, diabetic nephropathy, acid–base imbalance, and electrolyte disorder.^2^ The inflammatory processes mainly comprise the continuous release of pro‐inflammatory cytokines and oxidation stress damage.^3^ Secondly, dialysis procedures stimulate protein degradation and reduce protein synthesis; these responses persist following dialysis, which might lead to loss of muscle mass.^4^, ^5^

In patients undergoing dialysis, sarcopenia appears to confer adverse health outcomes, for example, functional decline, physical falls, hospitalization, and even death.^6^ Despite sarcopenia contributing to the poor prognosis of patients undergoing dialysis, the real clinical impact, especially related mortalities, has not been analysed. Furthermore, as the prevalence of sarcopenia in patients on dialysis has a wide range, its true impact on mortality is difficult to accurately ascertain.

One of the most important factors leading to the large variability in sarcopenia prevalence is the availability of different diagnostic criteria. There are more than four international recommended criteria, such as the diagnostic criteria developed by the European Working Group on Sarcopenia in Older People (EWGSOP),^7^, ^8^ Asian Working Group for Sarcopenia (AWGS),^9^, ^10^ Foundation for the National Institutes of Health Sarcopenia Project,^11^ and International Working Group on Sarcopenia.^12^

Future intervention researches for sarcopenia in dialysis patients need an accurate estimate of prevalence. However, the prevalence of sarcopenia is affected by the large variability in diagnostic criteria and characteristics of patients and varies too widely (4^13^–68%^14^) to use for comparison. Therefore, we performed this review to provide a comprehensive picture of the diagnostic criteria and prevalence of sarcopenia in those treated by dialysis and interpret its predictive value in terms of overall mortality. We also conducted subgroup analysis and meta‐regression (e.g. dialysis modality, age, and duration of dialysis) with an attempt to identify dialysis patients with high risk of sarcopenia; this endeavour could guide the selection of preventions for sarcopenia in dialysis patients.

## Methodology

### Inclusion and exclusion criteria

When performing this review, we followed the Preferred Reporting Items for Systematic Reviews and Meta‐Analyses 2020 statement (PRISMA 2020) principles,^15^ which is an updated set of guidelines from PRISMA 2009.^16^ To be included, a study was required to meet five specific criteria: (i) conducted on adults with ESRD who received dialysis treatment; (ii) provided prevalence data for sarcopenia in patients undergoing dialysis; (iii) defined sarcopenia as the presence of low muscle mass (LMM) plus low muscle strength (LMS), and/or low physical performance (LPP), or LMM alone; (iv) detected muscle mass with instruments commonly used previous to the study, for example, dual‐energy X‐ray absorptiometry, bioelectrical impedance analysis, magnetic resonance imaging, and bioelectrical impedance spectroscopy; and (v) study types were cross‐sectional or retrospective or prospective.

Studies were excluded based on the following criteria: (i) sarcopenia diagnostic criteria were not reported; (ii) animal studies, reports, editorials, reviews, comments, or conference abstracts; and (iii) published in languages other than English.

### Outcomes

The main outcomes of this review were (i) the methods used to diagnose sarcopenia, including diagnostic items, techniques for measurement, and sarcopenia threshold values, and (ii) sarcopenia prevalence in patients treated with dialysis and (iii) the impact of combined criteria (LMM plus LMS and/or LPP), LMM, LMS, and LPP on mortality in dialysis patients.

### Study databases and searching strategy

Using Ovid SP, we systematically screened relevant articles published up to 8 May 2021 from the databases Embase, MEDLINE, PubMed, and Cochrane Library without language restrictions. The detailed search strategy is shown in Supporting Information, *Table* S1. We also screened the citations included in the articles found in the database search for additional pertinent studies.

### Study selection

Two reviewers (X. S. and T. L.) screened the title and abstract of each search result independently while following the eligibility criteria to select possible studies for inclusion. Then, X. S. and T. L. separately reviewed the full text of these studies and decided on the final studies for inclusion. Lastly, X. S. and T. L. independently screened the citations used in the included papers to identify other studies meeting the search criteria. Any disagreement in study selection was resolved by discussing with a third, independent, reviewer (J. Y.). When the data used in two or more studies came from the same cohort, those with the largest sample size were included in the analysis.

### Data extraction

Two reviewers (X. S. and Y. Z.) extracted the data independently using standardized templates suitable for research objectives. A third assessor (J. Y.) reviewed the data‐extraction steps, and any disagreements were discussed and resolved. The following variables were collected using a data collection form: name of the first author, publication date, country, study design, sample size, proportion of men, mean age, dialysis method, duration of dialysis, diagnostic method for sarcopenia, skeletal muscle mass assessment technique, prevalence of sarcopenia, and sarcopenia diagnostic criteria. Meanwhile, if possible, the hazard ratio (HR) and 95% confidence interval (CI) for the overall mortality associated with the combined criteria (LMM plus LMS and/or LPP), LMM, LMS, and LPP were extracted.

### Assessing the risk of bias in the selected studies

The risk of bias in the studies selected for the review was separately estimated by X. S. and Y. Z. using the National Institutes of Health Quality Assessment Tool for Observational Cohort and Cross‐Sectional Studies—a validated procedure that assesses the quality of cohort and cross‐sectional studies^17^ that includes 14 items that are applied to estimate selection, information, and measurement biases as well as confounding. Additionally, we selected a tool that was designed specifically for assessing the risk of bias in prevalence studies and has been proved to have high interrater agreement.^18^ Ten items were used to address three domains of potential bias: measurement, selection, and analysis. A score of 8 or more was defined as low risk, 6 or 7 was considered moderate risk, and 5 or less was a high risk of bias. The arbitrator (J. Y.) was called upon to resolve disagreements among the reviewers.

### Data analyses

Statistical analyses were carried out with STATA/MP (Version 14.0, StataCorp, College Station, TX, USA), and forest plots were created to visualize the results. Heterogeneity was estimated by the *I* ^2^ test, with *I* ^2^ cut‐off values of 25%, 50%, and 75% respectively representing low, medium, and high heterogeneity.^19^ We applied a random‐effects model to calculate the pooled sarcopenia prevalence with a 95% CI when the *I* ^2^ index was interpreted as suggesting high heterogeneity; otherwise, the fixed‐effects model was used. In studies that evaluated sarcopenia by multiple diagnostic criteria, the prevalence most resembling the EWGSOP (2010) recommendation was pooled in the meta‐analysis. Additionally, to ascertain the impact of sarcopenia on mortality, the HR and 95% CI of the combined criteria (LMM plus LMS and/or LPP), LMM, LMS, and LPP were retrieved for meta‐analysis if possible. When we could extract HR and 95% CI from both univariate and multivariate analyses, the data from multivariate analyses were retrieved for meta‐analysis. Moreover, when both crude and adjusted HR and 95% CI were reported, adjusted HR and 95% CI were selected for data synthesis.

To investigate the possible reasons for heterogeneity, we performed subgroup analyses and meta‐regression on diagnostic criteria, dialysis modality, average age, and duration of dialysis. Sensitivity analysis was conducted to evaluate the quality and congruity of the results by deleting one study at a time. Publication bias was evaluated with the Egger test^20^ and Begg test^21^ (*P* < 0.05).

## Results

### Study selection

**Figure 1:**
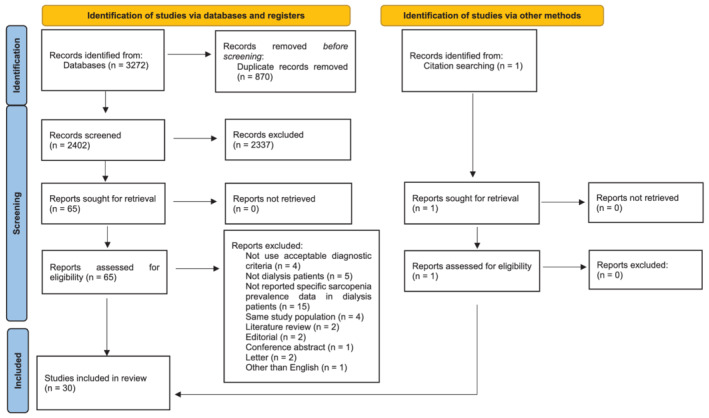
The flow chart of the literature selection.

*Figure* 1 depicts the flow chart of the literature selection process. In all, 3272 records were collated from the database search. After removing duplicates, 2402 titles and abstracts were screened, resulting in 65 relevant studies for full‐text screening, which resulted in 29 of these studies being included in our review. We also found an additional article after screening the reference lists of these 29 studies. The reasons used to exclude some articles subsequent to the full‐text screening were shown in the flow chart and *Table* S2. Finally, we selected 30 articles, involving 6162 participants, that satisfied with all inclusion criteria for the systematic review and meta‐analysis.

### Study characteristics

The characteristics of the included studies are summarized in *Table* 1. The 30^13^, ^14^, ^22^, ^23^, ^24^, ^25^, ^26^, ^27^, ^28^, ^29^, ^30^, ^31^, ^32^, ^33^, ^34^, ^35^, ^36^, ^37^, ^38^, ^39^, ^40^, ^41^, ^42^, ^43^, ^44^, ^45^, ^46^, ^47^, ^48^, ^49^ studies enrolled 6162 individuals who were included in the qualitative analysis. All of the included studies were published after 2013. Of these, 11 adopted a cross‐sectional design, 6 reported retrospective data, and 13 were prospective cohort studies. Twenty studies were conducted in HD populations, and 10 were conducted in PD populations. The mean age of the study participants ranged from 47.5 to 77.5 years, and the mean duration of dialysis ranged from 3 to 91.7 months. The included patients were sampled from a diversity of populations, including 14 studies conducted in Asia, 8 in America, and 8 in Europe (*Table* 1).

**Table 1:** Characteristics of the included studies and main findings

First author and year	Country	Study design	Sample size	Male, *n* (%)	Female, *n* (%)	Mean age (years)^a^	Dialysis method	Duration of dialysis (months)^a^	Prevalence of sarcopenia			Criteria (assessment method to detect sarcopenia)	HGS measure hand	Muscle mass measure time	Sarcopenia diagnostic criteria
									Total, *n* (%)	Male, *n* (%)	Female, *n* (%)				
Lamarca (2014)^22^	Brazil	Cross‐sectional	102	75	27	70.7	HD	27	13 (12.7%)	—	—	LMM (BIA) LMS (HGS)	Non‐fistula hand	After dialysis	EWGSOP (2010)
Isoyama (2014)^23^	Brazil	Prospective cohort	330	203	127	53	HD	—	66 (20.0%)	—	—	LMM (DXA) LMS (HGS)	Dominant hand or non‐fistula hand	After dialysis	EWGSOP (2010)
Ren (2016)^24^	China	Prospective observational	131	80	51	49.4	HD	71.3	18 (13.7%)	12 (15.0%)	6 (11.8%)	LMM (BIA) LMS (HGS)	Non‐fistula hand	Before dialysis	EWGSOP (2010)
Bataille (2017)^25^	France	Cross‐sectional	111	65	46	77.5	HD	28.4	35 (31.5%)	25 (38.5%)	10 (21.7%)	LMM (BIA) LMS (HGS)	Dominant hand	—	EWGSOP (2010)
Greenhall (2017)^26^	UK	Retrospective	490	—	—	55.3	PD	3	172 (35.1%)	—	—	LMM (BIA)	—	—	Others^b^
Jin (2017)^27^	China	Prospective cohort	117	57	60	60.8	PD	13.5	10 (8.6%)	—	—	LMM (BIA)	—	With peritoneal dialysate	Others^b^
Kamijo (2018)^28^	Japan	Prospective cohort	119	84	35	66.8	PD	—	13 (10.9%)	11 (13.1%)	2 (5.7%)	LMM (BIA) LMS (HGS) LPP (10mGS)	Dominant hand	—	AWGS (2014)
Kang (2017)^29^	Korea	Prospective cohort	631	341	290	53.2	PD	—	303 (48.0%)	175 (51.3%)	128 (44.1%)	LMM (DXA)	—	After drained out peritoneal dialysate	Others^b^
Kittiskulnam (2017)^30^	USA	Prospective cohort	645	378	267	56.7	HD	33.6	90 (14.0%)	53 (14.0%)	37 (13.9%)	LMM (BIS) LMS (HGS) LPP (4.6mGS)	Non‐fistula hand	Before dialysis	EWGSOP (2010)
Malhotra (2017)^31^	USA	Retrospective	122	76	46	47.5	HD	31	58 (47.5%)	47 (61.8%)	11 (23.9%)	LMM (DXA)		Non‐dialysis day	Others^b^
Abro (2018)^32^	UK	Retrospective	155	95	60	63.0	PD	9	17 (11.0%)	—	—	LMM (BIA) LMS (HGS)	Dominant hand	After drained out peritoneal dialysate	EWGSOP (2010)
As'habi (2018)^33^	Iran	Cross‐sectional	79	35	44	—	PD	—	9 (11.4%)	8 (22.9%)	1 (2.3%)	LMM (BIA) LMS (HGS) LPP (4mGS)	—	After drained out peritoneal dialysate	EWGSOP (2010)
Dierkes (2018)^34^	Norway	Cross‐sectional	24	17	7	63	HD	48	10 (41.7%)	—	—	LMM (BIA) LMS (HGS)	—	After dialysis	EWGSOP (2010)
Giglio (2018)^35^	Brazil	Prospective observational	170	111	59	70	HD	34.8	62 (36.5%)	52 (46.8%)	10 (16.9%)	LMM (DXA) + formula LMS (HGS)	Non‐fistula hand	After dialysis	EWGSOP (2010)
Lin (2018)^36^	China	Cross‐sectional	120	63	57	63.33	HD	56.52	20 (16.7%)	10 (15.9%)	10 (17.5%)	LMM (BIA) LMS (HGS) LPP (5mGS)	Non‐fistula hand	—	EWGSOP (2010)
Yoowannakul (2018)^37^	UK	Retrospective	600	373	227	66.3	HD	30.9	228 (38%)	154 (41.3%)	74 (32.6%)	LMM (BIA) LMS (HGS)	Dominant hand	After dialysis	EWGSOP (2010)
Yoowannakul (2018)^38^	UK	Retrospective	434	239	195	56	PD	3	205 (47.2%)	132 (55.2%)	73 (37.4%)	LMM (BIA)	—	After drained out peritoneal dialysate	Others^b^
Chiang (2019)^39^	USA	Prospective cohort	440	440	—	56.16	HD	32.4	75 (17.0%)	75 (17.0%)	—	LMM (BIS) LMS (HGS)	Both hands	Before dialysis	EWGSOP (2010)
Guida (2019)^43^	Italy	Cross‐sectional	88	59	29	53.4	PD	15.9	39 (44.3%)	22 (37.3%)	17 (58.6%)	LMM (BIA)	—	After drained out peritoneal dialysate	Others^b^
Kim (2019)^40^	Korea	Prospective observational	142	81	61	59.8	HD	50.23	47 (33.1%)	24 (29.6%)	23 (37.7%)	LMM (BIS) LMS (HGS)	Non‐fistula hand	—	EWGSOP (2010)
Lin (2020)^41^	China	Prospective cohort	126	65	61	63.2	HD	55.4	17 (13.5%)	9 (13.8%)	8 (13.1%)	LMM (BIS) LMS (HGS) LPP (6mGS)	Non‐fistula hand	Before dialysis	EWGSOP (2010)
Mori (2019)^42^	Japan	Prospective cohort	308	185	123	58.06	HD	77.3	124 (40.3%)	69 (37.3%)	55 (44.7%)	LMM (DXA) LMS (HGS)	Both hands	After dialysis	AWGS (2014)
da Silva (2019)^13^	Brazil	Cross‐sectional	50	24	26	55.74	PD	9.5	2 (4.0%)	—	—	LMM (DXA) LMS (HGS) LPP (4mGS)	—	—	EWGSOP (2010)
Kim (2020)^46^	Korea	Retrospective	160	109	51	55.1	PD	21.8	22 (13.8%)	—	—	LMM (BIS) LMS (HGS)	Dominant hand	—	Others^b^
Medeiros (2020)^44^	Brazil	Cross‐sectional	92	—	—	63.3	HD	—	50 (54.3%)	—	—	LMM (BIA) LMS (HGS) LPP (4mGS)	Non‐fistula hand	After dialysis	EWGSOP (2010)
Slee (2020)^45^	UK	Cross‐sectional	87	63	24	61.68	HD	65.9	38 (43.7%)	—	—	LMM (BIA) LMS (HGS)	—	After dialysis	EWGSOP (2010)
Song (2020)^47^	Korea	Prospective observational	88	50	38	60.6	HD	50.8	36 (40.9%)	—	—	LMM (BIS)	Non‐fistula hand	—	Others^b^
Matsuzawa (2021)^14^	Japan	Cross‐sectional	50	29	21	77.5	HD	38.5	34 (68%)	21 (72.4%)	13 (61.9%)	LMM (BIA) LMS (HGS) LPP (4mGS)	Both hands	After dialysis	AWGS (2019)
Miyazaki (2021)^48^	Japan	Cross‐sectional	20	14	6	76.5	HD	91.7	11 (55%)	—	—	LMM (DXA) LMS (HGS) LPP (6mGS)	—	—	AWGS (2019)
Takata (2021)^49^	Japan	Prospective observational	131	88	43	66.9	HD	—	8 (6.1%)	4 (4.5%)	4 (9.3%)	LMM (BIA)	—	—	Others^b^

AWGS, Asian Working Group for Sarcopenia; BIA, bioelectrical impedance analysis; BIS, bioelectrical impedance spectroscopy; DXA, dual‐energy X‐ray absorptiometry; EWGSOP, European Working Group on Sarcopenia in Older People; HGS, handgrip strength; LMM, lower muscle mass; LMS, lower muscle strength; LPP, lower physical performance.

^a^ Mean or median as reported.

^b^ Sarcopenia diagnostic criteria other than EWGSOP (2010), EWGSOP (2019), AWGS (2014), and AWGS (2019).

### Risk of bias in the included studies

The overall quality of included studies was moderate when assessed by the National Institutes of Health Quality Assessment Tool for Observational Cohort and Cross‐Sectional Studies^17^ (full details in *Figure* S1). Similarly, when appraised by prevalence studies assessment, 16 studies classed as moderate risk of bias studies and 14 classed as low risk of bias studies^18^ (*Figure* S2).

### Diagnostic method and prevalence of sarcopenia in dialysis patients

*Table* 2 summarized the commonly used diagnostic criteria of sarcopenia. Twenty‐two studies defined sarcopenia by LMM plus LMS and/or LPP, 17 of them defined sarcopenia by EWGSOP criteria, and 4 of them defined sarcopenia by AWGS criteria. Eight studies used only LMM to diagnose sarcopenia. Different measurement methods and cut‐off values were utilized to identify LMM, LMS, and LPP in these studies (*Table* 3). Muscle mass assessments were conducted with dual‐energy X‐ray absorptiometry (17 studies), bioelectrical impedance analysis (8 studies), and bioelectrical impedance spectroscopy (5 studies), and more than 10 cut‐off points were used to identify LMM. Muscle strength was assessed by handgrip dynamometry; the cut‐off points for identifying LMS varied from 16 to 20.7 kg in women and 26 to 36.6 kg in men. Physical performance was detected by walk tests with different walking distance, ranging from 4 to 10 m; the cut‐off points for identifying LPP were a gait speed <0.8 or <1.0 m/s.

**Table 2:** Diagnostic criteria of sarcopenia

	Low muscle mass (ASM)	Low muscle strength (HGS)	Lower physical performance (GS)	Sarcopenia diagnosis
AWGS (2014)	ASM/height^2^ <7.0 kg/m^2^ for men and <5.4 kg/m^2^ for women by using DXA ASM/height^2^ <7.0 kg/m^2^ for men and <5.7 kg/m^2^ for women by using BIA	HGS < 26 kg for men and <18 kg for women	Usual gait speed <0.8 m/s for both sexes	Sarcopenia: LMM plus LMS and/or LPP
AWGS (2019)	ASM/height^2^ <7.0 kg/m^2^ for men and <5.4 kg/m^2^ for women by using DXA ASM/height^2^ <7.0 kg/m^2^ for men and <5.7 kg/m^2^ for women by using BIA	HGS < 28 kg for men and <18 kg for women	Usual gait speed <1.0 m/s for both sexes	Possible sarcopenia: LMS or LPP Sarcopenia: LMM plus LMS or LPP Severe sarcopenia: LMM plus LMS and LPP
EWGSOP (2010)	ASM/height^2^ <7.26 kg/m^2^ for men and <5.5 kg/m^2^ for women	HGS < 30 kg for men and <20 kg for women	Usual gait speed ≤0.8 m/s for both sexes	Pre‐sarcopenia: LMM Sarcopenia: LMM plus LMS or LPP Severe sarcopenia: LMM plus LMS and LPP
EWGSOP (2019)	ASM/height^2^ <7.0 kg/m^2^ for men and <5.5 kg/m^2^ for women	HGS < 27 kg for men and <16 kg for women	Usual gait speed ≤0.8 m/s for both sexes	Possible sarcopenia: LMS Sarcopenia: LMM plus LMS Severe sarcopenia: LMM plus LMS and LPP
FINH	ALM/BMI < 0.789 for men and <0.512 for women	HGS < 26 kg for men and <16 kg for women	—	Sarcopenia: LMM plus LMS
IWGS	ASM/height^2^ <7.23 kg/m^2^ for men and <5.67 kg/m^2^ for women	—	Usual gait speed <1.0 m/s for both sexes	Sarcopenia: LMM plus LPP

ALM/BMI, appendicular lean mass/body mass index; ASM, appendicular skeletal muscle; AWGS, Asian Working Group for Sarcopenia; BIA, bioelectrical impedance analysis; DXA, dual‐energy X‐ray absorptiometry; EWGSOP, European Working Group on Sarcopenia in Older People; FNIH, Foundation for the National Institutes of Health Sarcopenia Project; GS, gait speed; HGS, handgrip strength; IWGS, International Working Group on Sarcopenia; LMM, lower muscle mass; LMS, lower muscle strength; LPP, lower physical performance.

**Table 3:** The details of diagnostic criteria and cut‐off points of each study

Low muscle mass		References
BIA	1. EWGSOP (2010) Janssen *et al*. (2004)^69^: SMI < 10.76 kg/m^2^ for men and <6.76 kg/m^2^ for women	Ren *et al*. (2016),^24^ Lin *et al*. (2018),^36^ As'habi *et al*. (2018),^33^ Lin *et al*. (2020),^41^ Medeiros *et al*. (2020),^44^ Slee *et al*. (2020),^45^ Malhotra *et al*. (2017),^31^ Greenhall *et al*. (2017)^26^
	2. EWGSOP (2010) Chien *et al*. (2008)^70^: MMI < 8.87 kg/m^2^ for men and <6.42 kg/m^2^ for women	Bataille *et al*. (2017),^25^ Dierkes *et al*. (2018)^34^
	3. EWGSOP (2010) Newman *et al*. (2003)^71^: ASMI < 7.23 kg/m^2^ for men and <5.67 kg/m^2^ for women	Yoowannakul *et al*. (2018),^37^ Abro *et al*. (2018)^32^
	4. EWGSOP (2010) Baumgartner *et al*. (1998)^72^: SMI ≥ 2 SD below sex‐specific means of healthy young adults	Yoowannakul *et al*. (2018)^38^
	5. EWGSOP (2010)^73^: LBMI > 2 SD below means of young individuals (men: <15.9 kg/m^2^; women: <12.8 kg/m^2^)	Lamarca *et al*. (2014)
	6. EWGSOP (2010) Janssen *et al*. (2002)^74^: SM/BW < 37% for men and <28% for women	Guida *et al*. (2019)^43^
	7. AWGS (2014 or 2019)^9^, ^10^: SMI < 7.0 kg/m^2^ for men and 5.7 kg/m^2^ for women	Jin *et al*. (2017),^27^ Kamijo *et al*. (2018),^28^ Matsuzawa *et al*. (2021),^14^ Takata *et al*. (2021)^49^
DXA	1. EWGSOP (2010) Baumgartner *et al*. (1998)^72^: ASMI < 7.3 kg/m^2^ in men and <5.5 kg/m^2^ in women	Isoyama *et al*. (2014),^23^ Giglio *et al*. (2018),^35^ da Silva *et al*. (2019)^13^
	2. AWGS (2014)^9^: SMI < 7.0 kg/m^2^ for men and 5.4 kg/m^2^ for women	Mori *et al*. (2019),^42^ Miyazaki *et al*. (2021)^48^
	3. FNIH^11^: ALM/BMI < 0.789 for men and <0.512 for women	Kang *et al*. (2017)^29^
BIS	1. EWGSOP (2010) Janssen *et al*. (2004)^69^: muscle mass of ≥2 SD below sex‐specific means of healthy young adults	Kittiskulnam *et al*. (2017),^30^ Chiang *et al*. (2019),^39^ Kim *et al*. (2019)^40^
	2. Marcelli *et al*. (2015)^75^: LTI below the 10th percentile of a reference population	Kim *et al*. (2020),^46^ Song *et al*. (2020)^47^

Low muscle strength		References
HGS	1. EWGSOP (2010) Lauretani *et al*. (2003)^76^: HGS < 30 kg for men and <20 kg for women	Isoyama *et al*. (2014),^23^ Ren *et al*. (2016),^24^ Bataille *et al*. (2017),^25^ Dierkes *et al*. (2018),^34^ Giglio *et al*. (2018),^35^ Yoowannakul *et al*. (2018),^37^ Lin *et al*. (2018),^36^ Abro *et al*. (2018),^32^ Kim *et al*. (2019),^40^ da Silva *et al*. (2019),^13^ Lin *et al*. (2020),^41^ Medeiros *et al*. (2020),^44^ Slee *et al*. (2020),^45^ Song *et al*. (2020)^47^
	2. AWGS (2014)^9^: HGS < 26 kg for men and <18 kg for women	Kamijo *et al*. (2018),^28^ As'habi *et al*. (2018),^33^ Chiang *et al*. (2019),^39^ Mori *et al*. (2019)^42^
	3. AWGS (2019)^10^: HGS < 28 kg for men and <18 kg for women	Miyazaki *et al*. (2021)^48^
	4. FINH^11^: HGS < 26 kg for men and <16 kg for women	Kittiskulnam *et al*. (2017)^30^
	5. Kim *et al*. (2018)^77^: HGS < 28.9 kg in men and <16.8 kg in women	Kim *et al*. (2020)^46^
	6. Schlüssel *et al*. (2008)^78^: HGS < 10th percentile of young individuals (men: right <36.6 kg and left <34.7 kg; women: right <20.7 kg and left <20.1 kg)	Lamarca *et al*. (2014)^22^

Lower physical performance		References
4mGS	1. AWGS (2014) and EWGSOP (2010) Lauretani *et al*.^76^: GS < 0.8 m/s	As'habi *et al*. (2018),^33^ da Silva *et al*. (2019),^13^ Medeiros *et al*. (2020),^44^ Matsuzawa *et al*. (2021)^14^
4.6mGS	1. FNIH^11^: walking speed <0.8 m/s	Kittiskulnam *et al*. (2017)^30^
5mGS	1. EWGSOP (2010) GS < 1.0 m/s	Lin *et al*. (2018)^36^
6mGS	1. EWGSOP (2010) GS < 0.8 m/s	Lin *et al*. (2020),^41^ Miyazaki *et al*. (2021)^48^
10mGS	1. AWGS (2014) GS < 0.8 m/s	Kamijo *et al*. (2018)^28^

ALM/BMI, appendicular lean mass/body mass index; ASMI, appendicular skeletal muscle index; AWGS, Asian Working Group for Sarcopenia; BIA, bioelectrical impedance analysis; BIS, bioelectrical impedance spectroscopy; DXA, dual‐energy X‐ray absorptiometry; EWGSOP, European Working Group on Sarcopenia in Older People; FNIH, Foundation for the National Institutes of Health Sarcopenia Project; GS, gait speed; HGS, handgrip strength; LBMI, lean body mass index; LTI, lean tissue index; MMI, muscle mass index; SD, standard deviation^79^; SM/BW, skeletal muscle/body weight; SMI, skeletal muscle mass index.

**Figure 2:**
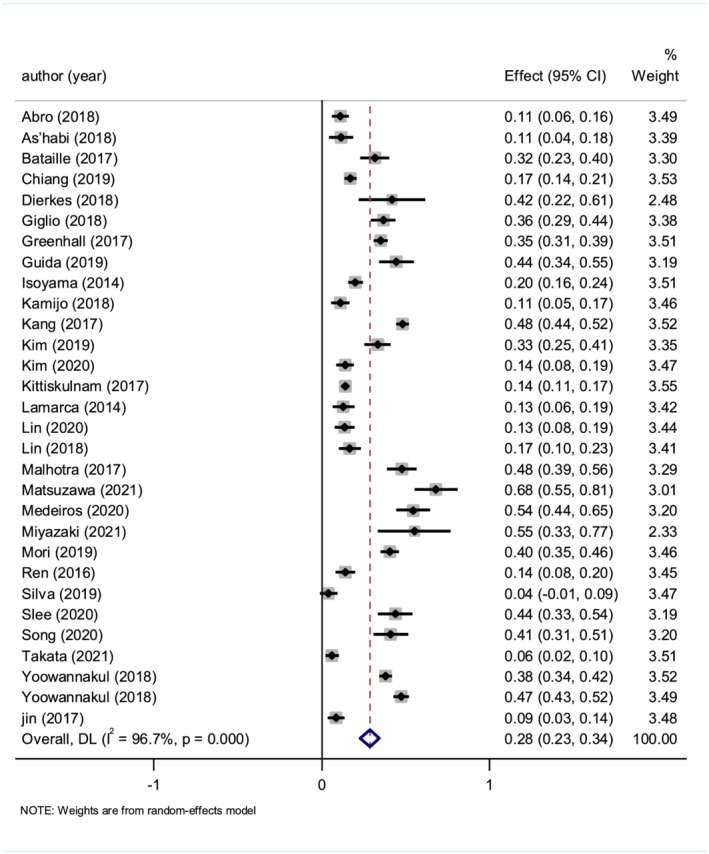
The pooled estimate prevalence of sarcopenia in dialysis patients.

In the 30 studies included, sarcopenia prevalence wide ranged from 4% to 68% (*Table* 1), and the pooled estimated prevalence was 28.5% (*I* ^2^ = 96.7%, 95% CI 22.9–34.1%; *Figure* 2).

### Subgroup analysis: sarcopenia definition, diagnostic criteria, and dialysis modality

The prevalence of sarcopenia in studies only using LMM [34.6% (*I* ^2^ = 98.1%, 95% CI 20.9–48.2%, 8 studies, 2101 cases)] were seemly higher than those defining sarcopenia using combined criteria [LMM plus LMS and/or LPP, 25.9% (*I* ^2^ = 94.9%, 95% CI 20.4–31.3%, 22 studies, 4061 cases)]; however, the difference did not exhibit statistical significance (*P* = 0.247, *Figure* S3). Additionally, the prevalence of sarcopenia defined by EWGSOP criteria [23.4% (*I* ^2^ = 94.2%, 95% CI 17.8–29.0%, 17 studies, 3404 cases)] was lower than the prevalence defined by AWGS criteria [42.6% (*I* ^2^ = 96.9%, 95% CI 18.7–66.6%, 4 studies, 497 cases)] and other criteria [32.2% (*I* ^2^ = 98.0%, 95% CI 19.6–44.8%, 9 studies, 2261 cases)]; however, the difference was not statistically significant (*P* = 0.165, *Figure* S3). Similarly, the prevalence of sarcopenia was revealed to be higher in HD populations [31% (*I* ^2^ = 95.5%, 95% CI 24.7–37.3%, 20 studies, 3839 cases)] than PD populations [23.4% (*I* ^2^ = 98.0%, 95% CI 11.9–34.9%, 10 studies, 2323 cases)]; however, the difference was not statistically significant (*P* = 0.255, *Figure* S3).

### Meta‐regression: average age and duration of dialysis

The pooled data on average age showed that it did not affect sarcopenia prevalence in the meta‐regression [regression coefficient 0.004 (95% CI: −0.005 to 0.012), *P* = 0.406, 29 studies, 6083 cases] (*Figure* S4). Additionally, the effect size could not be predicted using the duration of dialysis in the meta‐regression [regression coefficient 0.002 (95% CI −0.002 to 0.005), *P* = 0.327, 24 studies, 4780 cases] (*Figure* S5).

### Impact of combined criteria (low muscle mass plus low muscle strength and/or low physical performance), low muscle mass, and low muscle strength on mortality

Data from 10 studies were available to meta‐analyse mortality (*Table* 4). Patients who were diagnosed with sarcopenia by combined criteria (LMM plus LMS and/or LPP) had, on average, a higher risk of mortality than those without sarcopenia [HR 1.82 (*I* ^2^ = 26.3%, 95% CI 1.38–2.39), 6 studies, 1683 cases, *Figure* S6] and those diagnosed via LMM [HR 1.61 (*I* ^2^ = 26%, 95% CI 1.31–1.99), 8 studies, 2319 cases, *Figure* S7]. The fixed‐effect model was chosen because of the consistency between studies for the earlier associations. Moreover, patients with LMS were also confirmed to have a higher risk of mortality [HR 2.04 (*I* ^2^ = 80.4%, 95% CI 1.19–3.5), 6 studies, 1566 cases, *Figure* S8] than those possessing normal muscle strength. For the relationship between LMS and the risk of mortality, the random‐effects model was chosen, as suitable for the high between‐study heterogeneity (*I* ^2^ = 80.4%).

**Table 4:** The impact of sarcopenia on mortality in dialysis patients

First author and year	Univariate HR (95% CI)	Multivariate HR (95% CI) without adjustment	Multivariate HR (95% CI) with adjustment	Adjustment factors	Follow‐up time (months)^a^
Isoyama (2014)^23^			Combined criteria^b^: 1.93 (1.01–3.71) LMM: 1.23 (0.56–2.67) LMS: 1.98 (1.01–3.87)	Age, sex, diabetes, CVD, cholesterol, haemoglobin, GFR, and hs‐CRP	29
Kamijo (2018)^28^			LMM: 1.9 (0.74–4.89) LMS: 0.95 (0.77–1.17)	Age, gender, walking speed, SMI, grip strength, and CFS	19.6
Kang (2017)^29^	LMM: 1.74 (1.35–2.24)	LMM: 1.71 (1.28–2.26)			48
Kittiskulnam (2017)^30^			Combined criteria^b^: 1.65 (0.88–3.08) LMM: 1.70 (0.94–3.05) LMS: 1.68 (1.01–2.79) LPP: 2.25 (1.36–3.74)	Age, sex, race, co‐morbidities (diabetes mellitus, congestive heart failure, and coronary artery disease), and serum albumin	22.8
Malhotra (2017)^31^			LMM: 0.41 (0.15–1.1)	Age, gender, and sarcopenia obesity definitions	44
Giglio (2018)^35^		Combined criteria^b^: 2.02 (1.14–3.57) LMM: 1.49 (0.79–2.82) LMS: 2.03 (1.09–3.79)	Combined criteria^b^: 2.09 (1.05–4.20) LMM: 1.60 (0.73–3.53) LMS: 1.84 (0.92–3.68)	Age, gender, dialysis, vintage, and diabetes mellitus	36
Kim (2019)^40^			Combined criteria^b^: 6.99 (1.84–26.58) LMM: 2.77 (1.1–6.97) LMS: 5.65 (1.99–16.04)	Age, gender, BMI, *Kt*/*V*, albumin, diabetes, dialysis vintage, hs‐CRP, and previous history of coronary artery disease and cerebrovascular disease	51.6
Mori (2019)^42^		Combined criteria^b^: 1.31 (0.81–2.1)			76
Kim (2020)^46^			LMM: 1.98 (0.6–6.48) LMS: 2.97 (0.91–7.1)	Age, gender, BMI, dialysis duration, diabetes, serum level of albumin, CAD, and PAOD	24
Song (2020)^47^			Combined criteria^b^: 2.72 (1.11–6.63)	Age, gender, BMI, diabetes, CAD, CVD, PAOD, and dialysis vintage	62.4

BMI, body mass index; CAD, coronary artery disease; CFS, Clinical Frailty Scale; CI, confidence interval; CVD, cerebrovascular disease; GFR, glomerular filtration rate; HR, hazard ratio; hs‐CRP, high‐sensitivity C‐reactive protein; *Kt*/*V*, fractional clearance index for urea; LMM, lower muscle mass; LMS, lower muscle strength; LPP, lower physical performance; PAOD, peripheral artery occlusive disease; SMI, skeletal muscle index.

^a^ Mean or median as reported.

^b^ Combined criteria: LMM and either LMS or LPP.

### Publication bias and sensitivity analyses

No publication bias was detected in articles describing the prevalence of sarcopenia in patients on dialysis (Egger test: *P* = 0.092; Begg test: *P* = 0.054). Sensitivity analysis detected that the pooled prevalence of sarcopenia was not significantly affected by any individual study (*Figure* S9).

## Discussion

This review describes the sarcopenia prevalence in patients on dialysis and how it was affected by the various definitions of sarcopenia. The varied prevalence of sarcopenia may partly be explained by the variability of the definitions. However, there were no statistical differences between the different diagnostic items and different dialysis methods. Moreover, age and dialysis duration were not found to affect the prevalence of sarcopenia. Additionally, the pooled analyses indicated that combined criteria of sarcopenia (LMM plus LMS and/or LPP), LMM, and LMS in dialysis patients were all explicitly associated with an increased risk of mortality.

Because of exposure to long‐term conditions of oxidation stress and metabolic dysregulation, together with loss of nutrients into the dialysate, protein‐energy wasting (PEW)^50^, ^51^ presents universally in patients undergoing dialysis. Sarcopenia is both the main diagnostic criterion of PEW and an important manifestation of PEW, and its incidence is higher in dialysis patients. At present, the mechanism of sarcopenia in dialysis patients is not completely clear, but some mechanisms already explored include (i) chronic low‐grade inflammation: firstly, kidney function deteriorates, then oxidative stress is activated, uraemic toxins accumulate, and abnormally high levels of reactive oxygen species^52^ and uraemic toxins stimulate the occurrence of inflammatory reactions.^53^ Secondly, the decline of renal function results in a reduced capacity to excrete inflammatory factors, which leads to the persistence of the inflammatory response^3^; therefore, inflammatory cytokines, for example, tumour necrosis factor‐α, and interleukin‐6 and interleukin‐1, are often significantly increased in patients with ESRD,^54^, ^55^, ^56^ which increases the degradation and decreases the synthesis of muscle protein, resulting in muscle atrophy.^57^ (ii) Changes in hormone levels: an imbalance of hormone levels, presenting as decreasing levels of growth hormone, sex hormones (especially testosterone), insulin, and insulin‐like growth factor‐1,^58^ and increasing parathyroid hormone,^59^ glucocorticoids, and angiotensin II,^60^ and their interaction with the corresponding hormone receptor cause decreased protein synthesis and increased protein decomposition, eventually leading to the emergence of sarcopenia.^2^, ^5^ (iii) Changes in living status: reductions in appetite due to metabolic waste accumulation and some prescribed drugs, the imbalance of appetite‐regulating hormones, dietary restrictions, and gastrointestinal fullness may all lead to insufficient protein intake.^61^ Meanwhile, restricted activity during and fatigue after dialysis shortens the time taken for physical activity, which impairs muscle function. (iv) Protein loss during dialysis: both HD and PD procedures stimulate protein degradation and reduce protein synthesis, and these responses persist following dialysis,^4^, ^5^ also leading to loss of muscle mass. Of note, the mechanism of sarcopenia in dialysis patients is complicated and remains an aspect of research that merits consideration in the future.

Sarcopenia definitions used in diagnosing dialysis patients include LMM alone or combined criteria (LMM plus LMS and/or LPP), such as EWGSOP criteria, AWGS criteria, Foundation for the National Institutes of Health criteria, and International Working Group on Sarcopenia criteria. When only LMM was assessed, the pooled prevalence was estimated at 34.6%, while assessed combined criteria (LMM plus LMS and/or LPP) lowered it to 25.9%. The prevalence of sarcopenia presented an increasing trend in studies that used LMM alone, although the difference was not statistically significant. However, this merged result was lacking robustness caused by the high heterogeneity and small sample size of some studies among the included literature. For example, in two included articles that both diagnosed sarcopenia by combined criteria (LMM plus LMS and/or LPP), one reported the prevalence of sarcopenia in dialysis as 4%,^13^ while the value in the other was 68%,^14^ and both only involved 50 cases. The random‐effects model was applied in response to the high heterogeneity between the studies; however, the differences between the two groups became statistically significant when we implemented the fixed‐effects model, and this further proved that the merged result was not satisfactorily robust and the conclusion lacked reliability. At the same time, there was no statistical evidence of a difference between PD and HD populations, but sarcopenia seemed to be more prevalent in HD patients than PD patients. The present studies show that, compared with HD, PD has some advantages in preserving muscle mass and muscle function. Firstly, younger patients with ESRD who are in better physical condition are more likely to choose PD, as they are likely to have muscles of a better status. Secondly, compared with HD patients, PD‐treated patients have well‐preserved residual renal function and fewer complications.^62^ Additionally, PD is associated with better cognitive function^63^ and life quality^62^ than HD. All of these advantages of PD result in the conservation of muscle mass and muscle function.

Although sarcopenia has been traditionally seen as a condition associated with age, controversially, the age of the patients failed to demonstrate any clear influence on sarcopenia prevalence in our systematic review, implicating the importance of screening sarcopenia even in young dialysis patients. Of course, this relationship requires verification in studies with large dialysis cohorts. Moreover, the regression analysis suggested that the duration of dialysis had no significant effect on the incidence of sarcopenia. This result might relate to the fact that most of the included studies were interested in patients during the maintaining dialysis stage, whose physical condition tends to be relatively steady. As shown in the studies, physical function was in obvious decline for 3 months and mortality rates were consistently higher in the first 4 months after starting dialysis. However, the physical condition of dialysis patients is relatively steady in the maintaining dialysis stage, in which prolonged dialysis times may not have a significant effect on their physical condition.^64^, ^65^

In the general population, obesity is linked to a higher risk of cardiovascular disease and mortality. However, some studies found that elevated body mass index (BMI) was associated with improved survival in dialysis patients, which was described as the ‘obesity paradox’.^66^, ^67^, ^68^ The reason for this contradiction may lie in the decrease in BMI in dialysis patients that signifies the development or progression of sarcopenia, PEW, and cachexia, which have a clear association with poor prognoses in dialysis patients. The pooled analyses of 10 studies showed that the combined criteria of sarcopenia (LMM plus LMS and/or LPP) as well as LMM alone and LMS alone are strong mortality predictors. Compared with LMM, LMS was more robustly associated with mortality. Underpinning this, LMS is considered a component of severe sarcopenia. Because only one of the 10 studies measured LPP, we could not apply a meta‐analysis. However, this study indicated that the risk of death in dialysis patients with LPP is significantly increased. Therefore, for some settings in which it is difficult to measure multiple items of sarcopenia, such as ICUs, one or two of the items can have prognostic value.

To our best knowledge, this systematic review is the first to compare the diagnostic methods and prevalence of sarcopenia in dialysis patients. The study provides an up‐to‐date and accurate estimation of sarcopenia prevalence among the dialysis population, which is necessary in the calculation of sample size for future intervention studies in this arena. Additionally, in the randomized placebo‐controlled trials, the prevalence of the placebo group can compare with this meta‐analysis to ensure the placebo group has the expected prevalence. Meanwhile, understanding baseline patient characteristics that increase sarcopenia is critical for balanced randomization in interventional trials to prevent sarcopenia. Although the subgroup analysis did not find statistically significant population characteristics that increased the risk of sarcopenia, it was found that HD had a higher tendency to suffer from sarcopenia than PD. Moreover, it was found that sarcopenia was consistently associated with mortality in dialysis patients, which reinforces that the widespread and early clinical implementation of sarcopenia screening should help identify those at an increased risk of future health issues and help direct preventive therapies. For dialysis patients with sarcopenia, we should not only treat their medical disorders but also intervene in the progression of their sarcopenia to improve their prognoses and reduce their family and social healthcare burdens.

As with most studies, the design of this review was not without some limitations. Firstly, we only included literature released in English publications, which might have lent a selective bias to this review. Secondly, there was significant heterogeneity between the included studies in terms of the diagnostic methods, measurement approaches, and diagnostic thresholds, and so forth. Secondly, most of the included studies were cross‐sectional and had small cohorts, which can also lead to some between‐study heterogeneity. It is crucial, therefore, that future studies employ not only standardized methods of sarcopenia diagnosis but also adequate sample sizes to improve the quality of the original research. Thirdly, despite extracting adjusted estimates for multivariate analyses from the contributing studies, residual bias and confounding remain a possibility. Finally, although this review included studies from different continents (Asia, Europe, North America, and South America), data from Africa were not available, which limits its worldwide applicability.

## Conclusions

Sarcopenia is an important clinical condition shown to be prevalent in a clinically significant proportion of dialysis patients that is associated with a higher mortality risk. However, the clinical heterogeneity caused by the different diagnostic criteria, assessment procedures, and diagnostic thresholds for sarcopenia is substantial. Effective diagnostic criteria are key to the expeditious identification of sarcopenia in patients, and future longitudinal studies are needed to optimize management strategies aiming to improve individuals' lives and reduce family and social healthcare burdens.

## Author contributions

X.S. and J.Y. were involved in the study design, study protocol development, all analyses, and the management of all aspects of the systematic review. All co‐authors were involved in the literature search and participated in screening, full‐text reviewing, and data extracting. J.Y. provided advice on the analyses and aided in their interpretation. X.S. contributed to the writing of the final manuscript, and all co‐authors approved the final version for submission.

## Conflict of interest

None declared.

## Funding

This study was supported by grants from Chinese National Science & Technology Pillar Program (2020YFC2005600/02); Sichuan Province Science and Technology Support Program (2019YFS0277 and 2021YFS0136); 1·3·5 Project for Disciplines of Excellence—Clinical Research Incubation Project, West China Hospital, Sichuan University (19HXFH012); 1·3·5 Project for Disciplines of Excellence, West China Hospital, Sichuan University (ZYJC21005); and National Clinical Research Center for Geriatrics, West China Hospital, Sichuan University (Z20191003 and Z2018B13).

## Supporting information


Table S1. Search strategy by Embase, Medline, Pubmed, and Cochrane library via Ovid SP.
**Table S2.** The reasons for the exclusion of full‐text articles.
**Figure S1.** Risk of bias of the included studies using the National Institutes of Health Quality Assessment Tool for Observational Cohort and Cross‐Sectional Studies.
**Figure S2.** Risk of bias of the included studies using assessment tool explicitly for prevalence studies.
**Figure S3.** Prevalence of sarcopenia in dialysis patients according to different sarcopenia definition, diagnostic criteria, and dialysis modality.
**Figure S4.** Meta‐regression of the effect of average age on sarcopenia prevalence.
**Figure S5.** Meta‐regression of the effect of dialysis duration on sarcopenia prevalence.
**Figure S6.** Impact of combined criteria of sarcopenia (LMM plus LMS and/or LPP) on mortality in dialysis patients.
**Figure S7.** Impact of LMM on mortality in dialysis patients.
**Figure S8.** Impact of LMS on mortality in dialysis patients.
**Figure S9.** Sensitivity analysis.

## References

[1] Robinson BM , Akizawa T , Jager KJ , Kerr PG , Saran R , Pisoni RL . Factors affecting outcomes in patients reaching end‐stage kidney disease worldwide: differences in access to renal replacement therapy, modality use, and haemodialysis practices. Lancet 2016;388:294–306. 27226132 10.1016/S0140-6736(16)30448-2 PMC6563337

[2] Fahal IH . Uraemic sarcopenia: aetiology and implications. Nephrol Dial Transplant 2014;29:1655–1665. 23625972 10.1093/ndt/gft070

[3] Stenvinkel P , Alvestrand A . Inflammation in end‐stage renal disease: sources, consequences, and therapy. Semin Dial 2002;15:329–337. 12358637 10.1046/j.1525-139x.2002.00083.x

[4] Rajakaruna G , Caplin B , Davenport A . Peritoneal protein clearance rather than faster transport status determines outcomes in peritoneal dialysis patients. Perit Dial Int 2015;35:216–221. 25082839 10.3747/pdi.2013.00217 PMC4406317

[5] Wang XH , Mitch WE . Mechanisms of muscle wasting in chronic kidney disease. Nat Rev Nephrol 2014;10:504–516. 24981816 10.1038/nrneph.2014.112 PMC4269363

[6] Sabatino A , Cuppari L , Stenvinkel P , Lindholm B , Avesani CM . Sarcopenia in chronic kidney disease: what have we learned so far? J Nephrol 2021;34:1347–1372. 32876940 10.1007/s40620-020-00840-y PMC8357704

[7] Cruz‐Jentoft AJ , Baeyens JP , Bauer JM , Boirie Y , Cederholm T , Landi F , et al. Sarcopenia: European consensus on definition and diagnosis: Report of the European Working Group on Sarcopenia in Older People. Age Ageing 2010;39:412–423. 20392703 10.1093/ageing/afq034 PMC2886201

[8] Braun T . Sarcopenia: revised European consensus on definition and diagnosis. Phys Ther 2019;15:92.

[9] Chen L‐K , Liu L‐K , Woo J , Assantachai P , Auyeung T‐W , Bahyah KS , et al. Sarcopenia in Asia: consensus report of the Asian Working Group for Sarcopenia. J Am Med Dir Assoc 2014;15:95–101. 24461239 10.1016/j.jamda.2013.11.025

[10] Chen LK , Woo J , Assantachai P , Auyeung TW , Chou MY , Iijima K , et al. Asian Working Group for Sarcopenia: 2019 consensus update on sarcopenia diagnosis and treatment. J Am Med Dir Assoc 2020;21:300–7 e2. 32033882 10.1016/j.jamda.2019.12.012

[11] Studenski SA , Peters KW , Alley DE , Cawthon PM , McLean RR , Harris TB , et al. The FNIH Sarcopenia Project: rationale, study description, conference recommendations, and final estimates. J Gerontol A Biol Sci Med Sci 2014;69:547–558. 24737557 10.1093/gerona/glu010 PMC3991146

[12] Fielding RA , Vellas B , Evans WJ , Bhasin S , Morley JE , Newman AB , et al. Sarcopenia: an undiagnosed condition in older adults. Current consensus definition: prevalence, etiology, and consequences. J Am Med Dir Assoc 2011;12:249–256. 21527165 10.1016/j.jamda.2011.01.003 PMC3377163

[13] da Silva MZC , Vogt BP , do Carmo Reis NS , Teixeira Caramori JC . Update of the European consensus on sarcopenia: what has changed in diagnosis and prevalence in peritoneal dialysis? Eur J Clin Nutr 2019;73:1209–1211. 31300725 10.1038/s41430-019-0468-z

[14] Matsuzawa R , Yamamoto S , Suzuki Y , Imamura K , Harada M , Matsunaga A , et al. The clinical applicability of ultrasound technique for diagnosis of sarcopenia in hemodialysis patients. Clin Nutr 2021;40:1161–1167. 32798065 10.1016/j.clnu.2020.07.025

[15] Page MJ , McKenzie JE , Bossuyt PM , Boutron I , Hoffmann TC , Mulrow CD , et al. The PRISMA 2020 statement: an updated guideline for reporting systematic reviews. PLoS Med 2021;18:e1003583‐e. 33780438 10.1371/journal.pmed.1003583 PMC8007028

[16] Liberati A , Altman DG , Tetzlaff J , Mulrow C , Gotzsche PC , Ioannidis JPA , et al. The PRISMA statement for reporting systematic reviews and meta‐analyses of studies that evaluate health care interventions: explanation and elaboration. J Clin Epidemiol 2009;62:e1–e34. 19631507 10.1016/j.jclinepi.2009.06.006

[17] Quality assessment tool for observational cohort and cross‐sectional studies. 2015. https://www.nhlbi.nih.gov/health‐topics/study‐quality‐assessment‐tools

[18] Hoy D , Brooks P , Woolf A , Blyth F , March L , Bain C , et al. Assessing risk of bias in prevalence studies: modification of an existing tool and evidence of interrater agreement. J Clin Epidemiol 2012;65:934–939. 22742910 10.1016/j.jclinepi.2011.11.014

[19] Higgins JPT , Thompson SG , Deeks JJ , Altman DG . Measuring inconsistency in meta‐analyses. Br Med J 2003;327:557–560. 12958120 10.1136/bmj.327.7414.557 PMC192859

[20] Egger M , Smith GD , Schneider M , Minder C . Bias in meta‐analysis detected by a simple, graphical test. Bmj‐British Med J 1997;315:629–634. 10.1136/bmj.315.7109.629 PMC2127453 9310563

[21] Begg CB , Mazumdar M . Operating characteristics of a rank correlation test for publication bias. Biometrics 1994;50:1088–1101. 7786990

[22] Lamarca F , Carrero JJ , Rodrigues JCD , Bigogno FG , Fetter RL , Avesani CM . Prevalence of sarcopenia in elderly maintenance hemodialysis patients: the impact of different diagnostic criteria. J Nutr Health Aging 2014;18:710–717.2522611110.1007/s12603-014-0505-5

[23] Isoyama N , Qureshi AR , Avesani CM , Lindholm B , Barany P , Heimburger O , et al. Comparative associations of muscle mass and muscle strength with mortality in dialysis patients. Clin J Am Soc Nephrol 2014;9:1720–1728. 25074839 10.2215/CJN.10261013 PMC4186520

[24] Ren HQ , Gong DH , Jia FY , Xu B , Liu ZH . Sarcopenia in patients undergoing maintenance hemodialysis: incidence rate, risk factors and its effect on survival risk. Ren Fail 2016;38:364–371. 26738817 10.3109/0886022X.2015.1132173

[25] Bataille S , Serveaux M , Carreno E , Pedinielli N , Darmon P , Robert A . The diagnosis of sarcopenia is mainly driven by muscle mass in hemodialysis patients. Clin Nutr 2017;36:1654–1660. 27816311 10.1016/j.clnu.2016.10.016

[26] Greenhall GHB , Davenport A . Screening for muscle loss in patients established on peritoneal dialysis using bioimpedance. Eur J Clin Nutr 2017;71:70–75. 27782116 10.1038/ejcn.2016.202

[27] Jin S , Lu Q , Su C , Pang D , Wang T . Shortage of appendicular skeletal muscle is an independent risk factor for mortality in peritoneal dialysis patients. Perit Dial Int 2017;37:78–84. 27282855 10.3747/pdi.2016.00019

[28] Kamijo Y , Kanda E , Ishibashi Y , Yoshida M . Sarcopenia and frailty in PD: impact on mortality, malnutrition, and inflammation. Perit Dial Int 2018;38:447–454. 30065064 10.3747/pdi.2017.00271

[29] Kang SH , Cho KH , Park JW , Do JY . Low appendicular muscle mass is associated with mortality in peritoneal dialysis patients: a single‐center cohort study. Eur J Clin Nutr 2017;71:1405–1410. 28656967 10.1038/ejcn.2017.104

[30] Kittiskulnam P , Chertow GM , Carrero JJ , Delgado C , Kaysen GA , Johansen KL . Sarcopenia and its individual criteria are associated, in part, with mortality among patients on hemodialysis. Kidney Int 2017;92:238–247. 28318630 10.1016/j.kint.2017.01.024 PMC5483392

[31] Malhotra R , Deger SM , Salat H , Bian A , Stewart TG , Booker C , et al. Sarcopenic obesity definitions by body composition and mortality in the hemodialysis patients. J Ren Nutr 2017;27:84–90. 27876469 10.1053/j.jrn.2016.09.010 PMC5318243

[32] Abro A , Delicata LA , Vongsanim S , Davenport A . Differences in the prevalence of sarcopenia in peritoneal dialysis patients using hand grip strength and appendicular lean mass: depends upon guideline definitions. Eur J Clin Nutr 2018;72:993–999. 29921962 10.1038/s41430-018-0238-3

[33] As'habi A , Najafi I , Tabibi H , Hedayati M . Prevalence of sarcopenia and dynapenia and their determinants in Iranian peritoneal dialysis patients. Iran J Kidney Dis 2018;12:53–60. 29421778

[34] Dierkes J , Dahl H , Welland NL , Sandnes K , Saele K , Sekse I , et al. High rates of central obesity and sarcopenia in CKD irrespective of renal replacement therapy—an observational cross‐sectional study. BMC Nephrol 2018;19:259. 30305034 10.1186/s12882-018-1055-6 PMC6180401

[35] Giglio J , Kamimura MA , Lamarca F , Rodrigues J , Santin F , Avesani CM . Association of sarcopenia with nutritional parameters, quality of life, hospitalization, and mortality rates of elderly patients on hemodialysis. J Ren Nutr 2018;28:197–207. 29673501 10.1053/j.jrn.2017.12.003

[36] Lin YL , Liou HH , Lai YH , Wang CH , Kuo CH , Chen SY , et al. Decreased serum fatty acid binding protein 4 concentrations are associated with sarcopenia in chronic hemodialysis patients. Clin Chim Acta 2018;485:113–118. 29935964 10.1016/j.cca.2018.06.025

[37] Yoowannakul S , Tangvoraphonkchai K , Vongsanim S , Mohamed A , Davenport A . Differences in the prevalence of sarcopenia in haemodialysis patients: the effects of gender and ethnicity. J Hum Nutr Diet 2018;31:689–696. 29611250 10.1111/jhn.12555

[38] Yoowannakul S , Davenport A . Estimation of lean body mass by creatinine kinetics increases the prevalence of muscle wasting in peritoneal dialysis patients compared to bioimpedance. Eur J Clin Nutr 2018;72:1455–1457. 29330530 10.1038/s41430-017-0072-z

[39] Chiang JM , Kaysen GA , Segal M , Chertow GM , Delgado C , Johansen KL . Low testosterone is associated with frailty, muscle wasting and physical dysfunction among men receiving hemodialysis: a longitudinal analysis. Nephrol Dial Transplant 2019;34:802–810. 30085235 10.1093/ndt/gfy252 PMC6503299

[40] Kim JK , Kim SG , Oh JE , Lee YK , Noh JW , Kim HJ , et al. Impact of sarcopenia on long‐term mortality and cardiovascular events in patients undergoing hemodialysis. Korean J Intern Med 2019;34:599–607. 29161801 10.3904/kjim.2017.083 PMC6506738

[41] Lin YL , Liou HH , Wang CH , Lai YH , Kuo CH , Chen SY , et al. Impact of sarcopenia and its diagnostic criteria on hospitalization and mortality in chronic hemodialysis patients: a 3‐year longitudinal study. J Formos Med Assoc 2020;119:1219–1229. 31744647 10.1016/j.jfma.2019.10.020

[42] Mori K , Nishide K , Okuno S , Shoji T , Emoto M , Tsuda A , et al. Impact of diabetes on sarcopenia and mortality in patients undergoing hemodialysis. BMC Nephrol 2019;20:1–7. 30922266 10.1186/s12882-019-1271-8 PMC6437886

[43] Guida B , Trio R , Di Maro M , Memoli A , Di Lauro T , Belfiore A , et al. Prevalence of obesity and obesity‐associated muscle wasting in patients on peritoneal dialysis. Nutr Metab Cardiovas 2019;29:1390–1399. 10.1016/j.numecd.2019.05.057 31668791

[44] Medeiros MCW , Bandeira F , Henriques M , Silva NR , Farias MEB , Farias L . Serum sclerostin, body composition, and sarcopenia in hemodialysis patients with diabetes. Int J Nephrol 2020:4596920. 32095286 10.1155/2020/4596920 PMC7035555

[45] Slee A , McKeaveney C , Adamson G , Davenport A , Farrington K , Fouque D , et al. Estimating the prevalence of muscle wasting, weakness, and sarcopenia in hemodialysis patients. J Ren Nutr 2020;30:313–321. 31734056 10.1053/j.jrn.2019.09.004

[46] Kim C , Kim JK , Lee HS , Kim SG , Song YR . Longitudinal changes in body composition are associated with all‐cause mortality in patients on peritoneal dialysis. Clin Nutr 2020;30:30. 10.1016/j.clnu.2020.04.034 32451124

[47] Song YR , Kim JK , Lee HS , Kim SG , Choi EK . Serum levels of protein carbonyl, a marker of oxidative stress, are associated with overhydration, sarcopenia and mortality in hemodialysis patients. BMC Nephrol 2020;21:281. 32677905 10.1186/s12882-020-01937-z PMC7364609

[48] Miyazaki S , Iino N , Koda R , Narita I , Kaneko Y . Brain‐derived neurotrophic factor is associated with sarcopenia and frailty in Japanese hemodialysis patients. Geriatr Gerontol Int 2021;21:27–33. 33215785 10.1111/ggi.14089

[49] Takata T , Motoe A , Tanida K , Taniguchi S , Ida A , Yamada K , et al. Feasibility of computed tomography‐based assessment of skeletal muscle mass in hemodialysis patients. J Nephrol 2021;34:465–471. 32996109 10.1007/s40620-020-00871-5

[50] Obi Y , Qader H , Kovesdy CP , Kalantar‐Zadeh K . Latest consensus and update on protein‐energy wasting in chronic kidney disease. Curr Opin Clin Nutr Metab Care 2015;18:254–262. 25807354 10.1097/MCO.0000000000000171 PMC4506466

[51] Fouque D , Kalantar‐Zadeh K , Kopple J , Cano N , Chauveau P , Cuppari L , et al. A proposed nomenclature and diagnostic criteria for protein‐energy wasting in acute and chronic kidney disease. Kidney Int 2008;73:391–398. 18094682 10.1038/sj.ki.5002585

[52] Zhang L , Pan J , Dong Y , Tweardy DJ , Dong Y , Garibotto G , et al. Stat3 activation links a C/EBPδ to myostatin pathway to stimulate loss of muscle mass. Cell Metab 2013;18:368–379. 24011072 10.1016/j.cmet.2013.07.012 PMC3794464

[53] Watanabe H , Enoki Y , Maruyama T . Sarcopenia in chronic kidney disease: factors, mechanisms, and therapeutic interventions. Biol Pharm Bull 2019;42:1437–1445. 31474705 10.1248/bpb.b19-00513

[54] Schaap LA , Pluijm SMF , Deeg DJH , Harris TB , Kritchevsky SB , Newman AB , et al. Higher inflammatory marker levels in older persons: associations with 5‐year change in muscle mass and muscle strength. J Gerontol A Biol Sci Med Sci 2009;64:1183–1189. 19622801 10.1093/gerona/glp097 PMC2759573

[55] Mak RH , Ikizler TA , Kovesdy CP , Raj DS , Stenvinkel P , Kalantar‐Zadeh K . Wasting in chronic kidney disease (vol 2, pg 9, 2011). J Cachexia Sarcopeni 2011;2:119. 10.1007/s13539-011-0026-6 PMC3117998 22477651

[56] Axelsson J , Heimburger O , Stenvinkel P . Adipose tissue and inflammation in chronic kidney disease. Contrib Nephrol 2006;151:165–174. 16929140 10.1159/000095327

[57] Carrero JJ , Stenvinkel P , Cuppari L , Ikizler TA , Kalantar‐Zadeh K , Kaysen G , et al. Etiology of the protein‐energy wasting syndrome in chronic kidney disease: a consensus statement from the International Society of Renal Nutrition and Metabolism (ISRNM). J Ren Nutr 2013;23:77–90. 23428357 10.1053/j.jrn.2013.01.001

[58] Frost RA , Lang CH . Multifaceted role of insulin‐like growth factors and mammalian target of rapamycin in skeletal muscle. Endocrinol Metab Clin North Am 2012;41:297–322. 22682632 10.1016/j.ecl.2012.04.012 PMC3376019

[59] Kir S , Komaba H , Garcia AP , Economopoulos KP , Liu W , Lanske B , et al. PTH/PTHrP receptor mediates cachexia in models of kidney failure and cancer. Cell Metab 2016;23:315–323. 26669699 10.1016/j.cmet.2015.11.003 PMC4749423

[60] Morgan SA , Sherlock M , Gathercole LL , Lavery GG , Lenaghan C , Bujalska IJ , et al. 11 β‐Hydroxysteroid dehydrogenase type 1 regulates glucocorticoid‐induced insulin resistance in skeletal muscle. Diabetes 2009;58:2506–2515. 19675138 10.2337/db09-0525 PMC2768185

[61] Iorember FM . Malnutrition in chronic kidney disease. Front Pediatr 2018;6:161. 29974043 10.3389/fped.2018.00161 PMC6019478

[62] Merkus MP , Jager KJ , Dekker FW , Boeschoten EW , Stevens P , Krediet RT , et al. Quality of life in patients on chronic dialysis: self‐assessment 3 months after the start of treatment. The Necosad Study Group. Am J Kidney Dis 1997;29:584–592. 9100049 10.1016/s0272-6386(97)90342-5

[63] Neumann D , Mau W , Wienke A , Girndt M . Peritoneal dialysis is associated with better cognitive function than hemodialysis over a one‐year course. Kidney Int 2018;93:430–438. 29042081 10.1016/j.kint.2017.07.022

[64] Tamura MK , Covinsky KE , Chertow GM , Yaffe K , Landefeld CS , McCulloch CE . Functional status of elderly adults before and after initiation of dialysis. N Engl J Med 2009;361:1539–1547. 19828531 10.1056/NEJMoa0904655 PMC2789552

[65] Robinson BM , Zhang J , Morgenstern H , Bradbury BD , Ng LJ , McCullough KP , et al. Worldwide, mortality risk is high soon after initiation of hemodialysis. Kidney Int 2014;85:158–165. 23802192 10.1038/ki.2013.252 PMC3877739

[66] Kalantar‐Zadeh K , Abbott KC , Salahudeen AK , Kilpatrick RD , Horwich TB . Survival advantages of obesity in dialysis patients. Am J Clin Nutr 2005;81:543–554. 15755821 10.1093/ajcn/81.3.543

[67] Naderi N , Kleine C‐E , Park C , Hsiung J‐T , Soohoo M , Tantisattamo E , et al. Obesity paradox in advanced kidney disease: from bedside to the bench. Prog Cardiovasc Dis 2018;61:168–181. 29981348 10.1016/j.pcad.2018.07.001 PMC6131022

[68] Park J , Ahmadi S‐F , Streja E , Molnar MZ , Flegar KM , Gillen D , et al. Obesity paradox in end‐stage kidney disease patients. Prog Cardiovasc Dis 2014;56:415–425. 24438733 10.1016/j.pcad.2013.10.005 PMC4733536

[69] Janssen I , Baumgartner RN , Ross R , Rosenberg IH , Roubenoff R . Skeletal muscle cutpoints associated with elevated physical disability risk in older men and women. Am J Epidemiol 2004;159:413–421. 14769646 10.1093/aje/kwh058

[70] Chien M‐Y , Huang T‐Y , Wu Y‐T . Prevalence of sarcopenia estimated using a bioelectrical impedance analysis prediction equation in community‐dwelling elderly people in Taiwan. J Am Geriatr Soc 2008;56:1710–1715. 18691288 10.1111/j.1532-5415.2008.01854.x

[71] Newman AB , Kupelian V , Visser M , Simonsick E , Goodpaster B , Nevitt M , et al. Sarcopenia: alternative definitions and associations with lower extremity function. J Am Geriatr Soc 2003;51:1602–1609. 14687390 10.1046/j.1532-5415.2003.51534.x

[72] Baumgartner RN , Koehler KM , Gallagher D , Romero L , Heymsfield SB , Ross RR , et al. Epidemiology of sarcopenia among the elderly in New Mexico. Am J Epidemiol 1998;147:755–763. 9554417 10.1093/oxfordjournals.aje.a009520

[73] Kelly TL , Wilson KE , Heymsfield SB . Dual energy X‐ray absorptiometry body composition reference values from NHANES. Plos One 2009;4:e7038. 19753111 10.1371/journal.pone.0007038 PMC2737140

[74] Janssen I , Heymsfield SB , Ross R . Low relative skeletal muscle mass (sarcopenia) in older persons is associated with functional impairment and physical disability. J Am Geriatr Soc 2002;50:889–896. 12028177 10.1046/j.1532-5415.2002.50216.x

[75] Marcelli D , Usvyat LA , Kotanko P , Bayh I , Canaud B , Etter M , et al. Body composition and survival in dialysis patients: results from an international cohort study. Clin J Am Soc Nephrol 2015;10:1192–1200. 25901091 10.2215/CJN.08550814 PMC4491292

[76] Lauretani F , Russo CR , Bandinelli S , Bartali B , Cavazzini C , Di Iorio A , et al. Age‐associated changes in skeletal muscles and their effect on mobility: an operational diagnosis of sarcopenia. J Appl Physiol 2003;95:1851–1860. 14555665 10.1152/japplphysiol.00246.2003

[77] Kim CR , Jeon YJ , Kim MC , Jeong T , Koo WR . Reference values for hand grip strength in the South Korean population. PLoS ONE 2018;13:e0195485. 29624617 10.1371/journal.pone.0195485 PMC5889161

[78] Schlüssel MM , dos Anjos LA , de Vasconcellos MT , Kac G . Reference values of handgrip dynamometry of healthy adults: a population‐based study. Clin Nutr 2008;27:601–607. 18547686 10.1016/j.clnu.2008.04.004

[79] von Haehling S , Morley JE , Coats AJS , Anker SD . Ethical guidelines for publishing in the Journal of Cachexia, Sarcopenia and Muscle: update 2019. J Cachexia Sarcopenia Muscle 2019;10:1143–1145. 31661195 10.1002/jcsm.12501 PMC6818444

