# Supplementary material for: Diagnosis, prevalence, and mortality of sarcopenia in dialysis patients: a systematic review and meta‐analysis
Source: J Cachexia Sarcopenia Muscle. 2022 Jan 5;13(1):145–58. doi: 10.1002/jcsm.12890 (PMC8818609; doi:10.1002/jcsm.12890)
Supplement: Supplementary file 1 — Table S1. Search strategy by Embase, Medline, Pubmed, and Cochrane library via Ovid SP. Table S2. The reasons for the exclusion of full‐text articles. Figure S1. Risk of bias of the included studies using the National Institutes of Health Quality Assessment Tool for Observational Cohort and Cross‐Sectional Studies. Figure S2. Risk of bias of the included studies using assessment tool explicitly for prevalence studies. Figure S3. Prevalence of sarcopenia in dialysis patients according to different sarcopenia definition, diagnostic criteria, and dialysis modality. Figure S4. Meta‐regression of the effect of average age on sarcopenia prevalence. Figure S5. Meta‐regression of the effect of dialysis duration on sarcopenia prevalence. Figure S6. Impact of combined criteria of sarcopenia (LMM plus LMS and/or LPP) on mortality in dialysis patients. Figure S7. Impact of LMM on mortality in dialysis patients. Figure S8. Impact of LMS on mortality in dialysis patients. Figure S9. Sensitivity analysis. [file JCSM-13-145-s001.docx]

**Diagnosis, prevalence, and mortality of sarcopenia in patients undergoing dialysis: a systematic review and meta-analysis**

Table S1: Search strategy by Embase, Medline, Pubmed, and Cochrane library via Ovid SP

| 1. exp sarcopenia/ 2. (sarcopeni$ or myopeni$ or dynaponi$).tw. 3. ((muscle or muscular) adj2 (atroph$ or wasting$ or weak$ or loss$)).tw. 4. 1 or 2 or 3 5. exp hemodialysis/ 6. exp dialysis/ 7. (dialy$ or peritoneal dialy$ or hemodialy$ or haemodialy$ or hemodiafiltrat$ or haemodiafiltrat$ or hemofiltrat$ or haemofiltrat$ or intradialy$).kw,tw. 8. (end-stage kidney or end-stage renal or endstage kidney or endstage renal or stage 5 kidney or stage 5 renal).kw,tw. 9. (extracorporeal blood cleansing or renal replacement or artificial kidney).kw,tw. 10. (ESRF or ESKF or ESRD or ESKD).tw. 11. (PD or CAPD or CCPD or APD).tw. 12. 5 or 6 or 7 or 8 or 9 or 10 or 11 13. 4 and 12 |
| --- |

Table S2: The reasons for the exclusion of full-text articles

| Study | Reason for the exclusion |
| --- | --- |
| Moriyama (2019)^[1]^ | Not use acceptable diagnostic criteria: sarcopenia diagnosed by short physical performance battery (SPPB) |
| Lin (2019)^[2]^ | Not reported specific sarcopenia prevalence data in dialysis patients |
| Leonard (2019)^[3]^ | Not reported specific sarcopenia prevalence data in dialysis patients |
| Lai (2019)^[4]^ | Not dialysis patients: this article conducted in non-dialysis and dialysis chronic kidney disease patients |
| Kuki (2019)^[5]^ | Not reported specific sarcopenia prevalence data in dialysis patients |
| Giglio (2019)^[6]^ | Not dialysis patients: this article conducted in non-dialysis chronic kidney disease patients |
| Desai (2019)^[7]^ | Not reported specific sarcopenia prevalence data in dialysis patients |
| Chen (2019)^[8]^ | Not reported specific sarcopenia prevalence data in dialysis patients |
| Chan (2019)^[9]^ | Not reported specific sarcopenia prevalence data in dialysis patients |
| Bae (2019)^[10]^ | Exclusion study types: literature review |
| Aniort (2019)^[11]^ | Not reported specific sarcopenia prevalence data in dialysis patients |
| Yoowannakul (2018)^[12]^ | This article applied the same dataset with an included study^[13]^. |
| Tabibi (2018)^[14]^ | This article applied the same dataset with an included study^[15]^ |
| Marini (2018)^[16]^ | Not reported specific sarcopenia prevalence data in dialysis patients |
| 23 Kittiskulnam (2017)^[17]^ | This article applied the same dataset with an included study^[18]^. |
| Findlay (2017)^[19]^ | Exclusion study types: editorial |
| Morrell (2016)^[20]^ | Not reported specific sarcopenia prevalence data in dialysis patients |
| Chauveau (2016)^[21]^ | Other than English: French literature. |
| Kato (2014)^[22]^ | Not reported specific sarcopenia prevalence data in dialysis patients/ Exclusion study types: letter |
| Halle (2014)^[23]^ | Not use acceptable diagnostic criteria: sarcopenia diagnosed by mid-arm muscle circumference |
| Leal (2012)^[24]^ | Not reported specific sarcopenia prevalence data in dialysis patients |
| Kaysen (2005)^[25]^ | Exclusion study types: editorial |
| Tangvoraphonkchai (2018)^[26]^ | Not use acceptable diagnostic criteria: sarcopenia diagnosed by handgrip strength |
| Kang (2013)^[27]^ | Not reported specific sarcopenia prevalence data in dialysis patients |
| Park (2019)^[28]^ | Exclusion study types: conference abstract |
| Garibotto (2019)^[29]^ | Exclusion study types: review |
| Hung (2017)^[30]^ | Not reported specific sarcopenia prevalence data in dialysis patients |
| Wilkinson (2020)^[31]^ | Not dialysis patients: this article conducted in non-dialysis and dialysis chronic kidney disease patients |
| FiLon (2019)^[32]^ | Not reported specific sarcopenia prevalence data in dialysis patients |
| Do (2020)^[33]^ | Not reported specific sarcopenia prevalence data in dialysis patients |
| Moon (2015)^[34]^ | Not dialysis patients: this article conducted in non-dialysis chronic kidney disease patients |
| Hotta (2015)^[35]^ | Exclusion study types: letter |
| Kim (2014)^[36]^ | This article applied the same dataset with an included study^[37]^. |
| Lopes (2019)^[38]^ | Not reported specific sarcopenia prevalence data in dialysis patients |
| Marini (2020)^[39]^ | Not use acceptable diagnostic criteria: sarcopenia diagnosed by the Strength, Assistance with walking, Rise from a Chair, Climb stairs and Falls (SARC-F) |
| Zhou (2021)^[40]^ | Not dialysis patients: this article conducted in non-dialysis chronic kidney disease patients |

Figure S1: Risk of bias of the included studies using the National Institutes of Health Quality Assessment Tool for Observational Cohort and Cross-Sectional Studies.

Yes = criteria met No = criteria not met NA = not applicable NR = not reported

Criteria

Q1. Was the research question or objective in this paper clearly stated?

Q2. Was the study population clearly specified and defined?

Q3. Was the participation rate of eligible persons at least 50%?

Q4. Were all the subjects selected or recruited from the same or similar populations (including the same time period)? Were inclusion and exclusion criteria for being in the study prespecified and applied uniformly to all participants?

Q5. Was a sample size justification, power description, or variance and effect estimates provided?

Q6. For the analyses in this paper, were the exposure(s) of interest measured prior to the outcome(s) being measured?

Q7. Was the timeframe sufficient so that one could reasonably expect to see an association between exposure and outcome if it existed?

Q8. For exposures that can vary in amount or level, did the study examine different levels of the exposure as related to the outcome (e.g., categories of exposure, or exposure measured as continuous variable)?

Q9. Were the exposure measures (independent variables) clearly defined, valid, reliable, and implemented consistently across all study participants?

Q10. Was the exposure(s) assessed more than once over time?

Q11. Were the outcome measures (dependent variables) clearly defined, valid, reliable, and implemented consistently across all study participants?

Q12. Were the outcome assessors blinded to the exposure status of participants?

Q13. Was loss to follow-up after baseline 20% or less?

Q14. Were key potential confounding variables measured and adjusted statistically for their impact on the relationship between exposure(s) and outcome(s)?

Figure S2: Risk of bias of the included studies using assessment tool explicitly for prevalence studies

Low = low risk of bias High = high risk of bias Moderate = moderate risk of bias

Criteria

External validity :

Q1. Was the study’s target population a close representation of the national population in relation to relevant variables?

Q2. Was the sampling frame a true or close representation of the target population?

Q3. Was some form of random selection used to select the sample, OR was a census undertaken?

Q4. Was the likelihood of nonresponse bias minimal?

Internal validity :

Q5. Were data collected directly from the subjects (as opposed to a proxy)?

Q6. Was an acceptable case definition used in the study?

Q7. Was the study instrument that measured the parameter of interest shown to have validity and reliability?

Q8. Was the same mode of data collection used for all subjects?

Q9. Was the length of the shortest prevalence period for the parameter of interest appropriate?

Q10. Were the numerator(s) and denominator(s) for the parameter of interest appropriate?

Q11. Summary item on the overall risk of study bias.

Figure **S3**: **Prevalence of sarcopenia in dialysis patients according to different sarcopenia definition, diagnostic criteria, and dialysis modality.**

Heterogeneity between groups: p = 0.664

Overall, DL (I

2

= 96.9%, p = 0.000)

Subgroup, DL (I

2

= 97.7%, p = 0.000)

PD

HD

dialysis modality

Subgroup, DL (I

2

= 98.0%, p = 0.000)

1 criteria

>1 criteria

diagnostic criteria

Subgroup, DL (I

2

= 98.0%, p = 0.000)

**others**

**AWGS**

**EWGSOP**

sarcopenia definition

subgroup

group and

2323

3839

2101

4061

2261

497

3404

10

20

8

22

9

4

17

0.30 (0.26, 0.34)

0.27 (0.20, 0.35)

0.23 (0.22, 0.25)

0.31 (0.30, 0.32)

0.30 (0.22, 0.39)

0.35 (0.33, 0.37)

0.26 (0.25, 0.27)

0.33 (0.24, 0.41)

0.32 (0.30, 0.34)

0.43 (0.38, 0.47)

0.23 (0.22, 0.25)

Effect (95% CI)

total

study

0

.5

NOTE: Weights and between-subgroup heterogeneity test are from random-effects model

Figure **S4**: Meta-regression of the effect of average age on sarcopenia prevalence.

0

.2

.4

.6

.8

p

50

60

70

80

age

Regression coefficient 0.004 (95%CI -0.005 to 0.012), P = 0.406. Circle diameters reflective of proportional study sample size.

Figure **S5**: Meta-regression of the effect of dialysis duration on sarcopenia prevalence.

0

.2

.4

.6

.8

p

0

20

40

60

80

100

duration

Regression coefficient 0.002 (95%CI -0.002 to 0.005), P = 0.327. Circle diameters reflective of proportional study sample size.

Figure **S6**: Impact of **combined criteria of sarcopenia (LMM plus LMS and/or LPP)** on mortality in dialysis patients.

Figure **S7**: Impact of LMM on mortality in dialysis patients

Figure **S8**: Impact of LMS on mortality in dialysis patients

Figure **S9**: Sensitivity analysis

**Reference:**

1. Moriyama Y, Hara M, Aratani S, Ishikawa H, Kono K, Tamaki M. The association between six month intra-dialytic resistance training and muscle strength or physical performance in patients with maintenance hemodialysis: a multicenter retrospective observational study. BMC Nephrol. 2019;20:172.

2. Lin YL, Chen SY, Lai YH, Wang CH, Kuo CH, Liou HH, et al. Angiotensin II receptor blockade is associated with preserved muscle strength in chronic hemodialysis patients. BMC Nephrol. 2019;20:54.

3. Leonard MB, Wehrli FW, Ziolkowski SL, Billig E, Long J, Nickolas TL, et al. A multi-imaging modality study of bone density, bone structure and the muscle - bone unit in end-stage renal disease. Bone. 2019;127:271-9.

4. Lai S, Muscaritoli M, Andreozzi P, Sgreccia A, De Leo S, Mazzaferro S, et al. Sarcopenia and cardiovascular risk indices in patients with chronic kidney disease on conservative and replacement therapy. Nutrition. 2019;62:108-14.

5. Kuki A, Tanaka K, Kushiyama A, Tanaka Y, Motonishi S, Sugano Y, et al. Association of gait speed and grip strength with risk of cardiovascular events in patients on haemodialysis: a prospective study. BMC Nephrol. 2019;20:196.

6. Giglio J, Kamimura MA, Souza NC, Bichels AV, Cordeiro AC, Pinho N, et al. Muscle mass assessment by computed tomography in chronic kidney disease patients: agreement with surrogate methods. Eur J Clin Nutr. 2019;73:46-53.

7. Desai M, Mohamed A, Davenport A. A pilot study investigating the effect of pedalling exercise during dialysis on 6-min walking test and hand grip and pinch strength. Int J Artif Organs. 2019;42:161-6.

8. Chen SC, Chung WS, Wu PY, Huang JC, Chiu YW, Chang JM, et al. Associations among Geriatric Nutrition Risk Index, bone mineral density, body composition and handgrip strength in patients receiving hemodialysis. Nutrition. 2019;65:6-12.

9. Chan KN, Chen Y, Lit Y, Massaband P, Kiratli J, Rabkin R, et al. A randomized controlled trial of exercise to prevent muscle mass and functional loss in elderly hemodialysis patients: Rationale, study design, and baseline sample. Contemp Clin Trials Commun. 2019;15:100365.

10. Bae EH. Is sarcopenia a real risk factor for mortality in patients undergoing hemodialysis? Korean J Intern Med. 2019;34:507-9.

11. Aniort J, Stella A, Philipponnet C, Poyet A, Polge C, Claustre A, et al. Muscle wasting in patients with end-stage renal disease or early-stage lung cancer: common mechanisms at work. J Cachexia Sarcopenia Muscle. 2019;10:323-37.

12. Yoowannakul S, Tangvoraphonkchai K, Davenport A. The prevalence of muscle wasting (sarcopenia) in peritoneal dialysis patients varies with ethnicity due to differences in muscle mass measured by bioimpedance. Eur J Clin Nutr. 2018;72:381-7.

13. Yoowannakul S, Davenport A. Estimation of lean body mass by creatinine kinetics increases the prevalence of muscle wasting in peritoneal dialysis patients compared to bioimpedance. Eur J Clin Nutr. 2018;72:1455-7.

14. Tabibi H, As'habi A, Najafi I, Hedayati M. Prevalence of dynapenic obesity and sarcopenic obesity and their associations with cardiovascular disease risk factors in peritoneal dialysis patients. Kidney Res Clin Pract. 2018;37:404-13.

15. As'habi A, Najafi I, Tabibi H, Hedayati M. Prevalence of Sarcopenia and Dynapenia and Their Determinants in Iranian Peritoneal Dialysis Patients. Iran J Kidney Dis. 2018;12:53-60.

16. Marini AC, Motobu RD, Freitas ATV, Laviano A, Pimentel GD. Pre-sarcopenia in patients undergoing hemodialysis: Prevalence and association with biochemical parameters. Clin Nutr ESPEN. 2018;28:236-8.

17. Kittiskulnam P, Chertow GM, Carrero JJ, Delgado C, Kaysen GA, Johansen KL. Sarcopenia and its individual criteria are associated, in part, with mortality among patients on hemodialysis. Kidney Int. 2017;92:238-47.

18. Kittiskulnam P, Carrero JJ, Chertow GM, Kaysen GA, Delgado C, Johansen KL. Sarcopenia among patients receiving hemodialysis: weighing the evidence. J Cachexia Sarcopeni. 2017;8:57-68.

19. Findlay MD, Mark PB. Reduced and declining physical function in prevalent dialysis patients-identifying the vulnerable. Age Ageing. 2017;46:541-3.

20. Morrell GR, Ikizler TA, Chen X, Heilbrun ME, Wei G, Boucher R, et al. Psoas Muscle Cross-sectional Area as a Measure of Whole-body Lean Muscle Mass in Maintenance Hemodialysis Patients. J Ren Nutr. 2016;26:258-64.

21. Chauveau P, Moreau K, Lasseur C, Fouque D, Combe C, Aparicio M. [Sarcopenia or uremic myopathy in CKD patients]. Nephrol Ther. 2016;12:71-5.

22. Kato A, Takita T, Kumagai H. Relationship between arterial stiffening and skeletal muscle atrophy in hemodialysis patients: a gender comparative study. J Cachexia Sarcopenia Muscle. 2014;5:247-9.

23. Halle MP, Zebaze PN, Mbofung CM, Kaze F, Mbiatat H, Ashuntantang G, et al. Nutritional status of patients on maintenance hemodialysis in urban sub-Saharan Africa: evidence from Cameroon. J Nephrol. 2014;27:545-53.

24. Leal VO, Moraes C, Stockler-Pinto MB, Lobo JC, Farage NE, Velarde LG, et al. Is a body mass index of 23 kg/m(2) a reliable marker of protein-energy wasting in hemodialysis patients? Nutrition. 2012;28:973-7.

25. Kaysen GA. Diabetes, a cause of progressive sarcopenia in dialysis patients? Kidney Int. 2005;68:2396-7.

26. Tangvoraphonkchai K, Hung R, Sadeghi-Alavijeh O, Davenport A. Differences in Prevalence of Muscle Weakness (Sarcopenia) in Haemodialysis Patients Determined by Hand Grip Strength Due to Variation in Guideline Definitions of Sarcopenia. Nutr Clin Pract. 2018;33:255-60.

27. Kang SH, Park JW, Yoon KW, Do JY. Limb/trunk lean mass ratio as a risk factor for mortality in peritoneal dialysis patients. J Ren Nutr. 2013;23:315-23.

28. Park HC, Jung KS, Kim SH, Lee JE, Kim H, Choi HY, et al. Sat-065 Low Natural Killer Cell Activity May Contribute to Development of Sarcopenia in Hemodialysis Patients. Kidney International Reports. 2019;4:S30. doi:10.1016/j.ekir.2019.05.089

29. Garibotto G, Picciotto D, Verzola D. Testosterone deficiency, frailty and muscle wasting in CKD: a converging paradigm? Nephrol Dial Transplant. 2019;34:723-6.

30. Hung R, Wong B, Goldet G, Davenport A. Differences in Prevalence of Muscle Wasting in Patients Receiving Peritoneal Dialysis per Dual-Energy X-Ray Absorptiometry Due to Variation in Guideline Definitions of Sarcopenia. Nutrition in Clinical Practice. 2017;32:539-44.

31. Wilkinson TJ, Nixon DGD, Richler-Potts D, Neale J, Song Y, Smith AC. Identification of the most clinically useful skeletal muscle mass indices pertinent to sarcopenia and physical performance in chronic kidney disease. Nephrology. 2020;25:467-74.

32. FiLon T, Rogowski L, Kusztal M, Bulinska K, Pawlaczyk W, GoLebiowski T, et al. Muscle strength and bone mass density in haemodialysis patients. Physiotherapy Quarterly. 2019;27:39-45.

33. Do JY, Kang SH. Association Between Peritonitis and Low Muscle Mass in Peritoneal Dialysis Patients. Journal of Renal Nutrition. 2020;30:341-6.

34. Moon SJ, Kim TH, Yoon SY, Chung JH, Hwang HJ. Relationship between stage of chronic kidney disease and sarcopenia in Korean aged 40 years and older using the Korea National Health and Nutrition Examination Surveys (KNHANES IV-2, 3, and V-1, 2), 2008-2011. PLoS ONE. 2015;10 (6) (no pagination):

35. Hotta C, Hiraki K, Wakamiya A, Otobe Y, Watanabe S, Izawa KP, et al. Relation of physical function and physical activity to sarcopenia in hemodialysis patients: A preliminary study. International Journal of Cardiology. 2015;191:198-200.

36. Kim JK, Choi SR, Choi MJ, Kim SG, Lee YK, Noh JW, et al. Prevalence of and factors associated with sarcopenia in elderly patients with end-stage renal disease. Clinical Nutrition. 2014;33:64-8.

37. Kim JK, Kim SG, Oh JE, Lee YK, Noh JW, Kim HJ, et al. Impact of sarcopenia on long-term mortality and cardiovascular events in patients undergoing hemodialysis. Korean J Intern Med. 2019;34:599-607.

38. Lopes LCC, Mota JF, Prestes J, Schincaglia RM, Silva DM, Queiroz NP, et al. Intradialytic Resistance Training Improves Functional Capacity and Lean Mass Gain in Individuals on Hemodialysis: a Randomized Pilot Trial. Archives of physical medicine and rehabilitation. 2019;100:2151-8.

39. Marini ACB, Perez DRS, Fleuri JA, Pimentel GD. SARC-F Is Better Correlated with Muscle Function Indicators than Muscle Mass in Older Hemodialysis Patients. Journal of Nutrition, Health & Aging. 2020;24:999-1002.

40. Zhou Y, Hellberg M, Hellmark T, Hoglund P, Clyne N. Muscle mass and plasma myostatin after exercise training: a substudy of Renal Exercise (RENEXC)-a randomized controlled trial. Nephrol Dial Transplant. 2021;36:95-103.
